# Ambulatory Care Sensitive Conditions Encountered by a Homeless Healthcare Team

**DOI:** 10.1007/s11606-024-09092-z

**Published:** 2024-12-03

**Authors:** Evan Michael Shannon, Un Young Chung, Marjan Javanbakht, Medell Briggs-Malonson, Catherine Weaver, Brian Zunner-Keating, Sae Takada

**Affiliations:** 1 Division of General Internal Medicine and Health Services Research, UCLA David Geffen School of Medicine, Los Angeles, CA USA; 2 Department of Epidemiology, UCLA Fielding School of Public Health, Los Angeles, CA USA; 3 UCLA Health, Los Angeles, CA USA; 4 Department of Emergency Medicine, UCLA David Geffen School of Medicine, Los Angeles, CA USA

## INTRODUCTION

Ambulatory care sensitive conditions (ACSCs) are a set of diagnoses that can be treated in ambulatory settings to prevent hospitalizations. Hospitalizations for ACSCs are a validated proxy measure for access to and quality of ambulatory care.^1^ People experiencing homelessness (PEH) face barriers accessing ambulatory care and chronic disease self-management.^2^ These factors place them at higher risk for hospitalizations for ACSCs and contribute to high rates of acute care utilization in this population.^3,4^

Field-based homeless healthcare teams provide health and social services to address the unique needs and circumstances of PEH.^5^ In this model, medical practitioners care for patients in encampments, shelters, and transitional housing sites as opposed to brick-and-mortar clinics, which removes some barriers to healthcare access. Field-based homeless healthcare teams can diagnose and treat ACSCs among PEH.^4^ To date, there is limited literature on the frequency of ACSCs encountered by field-based homeless healthcare teams. We aimed to describe the experience of a Los Angeles County homeless healthcare team with ACSCs and determine if certain sociodemographic subgroups are disproportionately affected by ACSCs.

## METHODS

We performed a cross-sectional analysis of adult patients (18 years old or older) evaluated by the Homeless Healthcare Collaborative (HHC), UCLA Health’s field-based homeless healthcare team. HHC provides free, trauma-informed health care, including preventative and urgent care, and social services to PEH in Los Angeles County. We examined encounters between HHC’s rollout in January 2022 through September 2023. We abstracted data from the electronic health records (EHR). This study was certified exempt by the UCLA Institutional Review Board.

The outcome of interest was the presence of a diagnosis of one or more ACSCs during a clinical encounter: anemia, angina, asthma, cellulitis, congestive heart failure, chronic obstructive pulmonary disease (COPD), dental condition, diabetes mellitus, epilepsy, gangrene, gastroenteritis, hypertension, lower extremity ulcer, pneumonia, and urinary tract infection. Diagnoses were identified based on International Classification of Diseases, Tenth Revision (ICD-10) codes. Sociodemographic factors including age, sex, and race and ethnicity for each patient were extracted from the EHR. We performed descriptive statistics overall and by ACSC status. Factors associated with ACSCs were evaluated using multi-level multivariable logistic regression model which accounted for repeat visits for some patients.

## RESULTS

HHC performed 5,520 encounters among 3,226 unique patients. Overall, ACSCs were present in 1,706 (30.9%) encounters among 1,034 (32.1%) unique patients (Table 1). The most common ACSCs encountered were hypertension (796; 46.6%), cellulitis (347; 20.3%), diabetes (277; 16.2%), asthma (112; 6.6%), dental condition (107; 6.3%), and COPD (84; 4.9%). Based on multivariable analyses, the odds of having an encounter with an ACSC increased with age (adjusted OR 1.16 per age increase by 10 years, 95% CI 1.09, 1.25; p < 0.001) and differed by race and ethnicity with non-Hispanic Black patients having higher odds of ACSCs as compared to non-Hispanic White patients (adjusted OR 1.27, 95% CI 1.01, 1.61; p = 0.042) (Table 2).

**Table 1:** Characteristics of Patients with Encounters Including and Not Including Ambulatory Care Sensitive Conditions (ACSCs)

Characteristic, *n* (%)	All	ACSC	No ACSC	*p*-value^*^
Total	3226	1034 (32.1)	2192 (67.9)	
Age^†^				< 0.001
18–39	829 (25.7)	183 (17.7)	646 (29.5)	
40–65	1989 (61.7)	700 (67.7)	1289 (58.8)	
> 65	408 (12.7)	151 (14.6)	257 (11.7)	
Gender				0.62
Female	1064 (33.0)	335 (32.4)	729 (33.3)	
Male	2155 (66.8)	697 (67.5)	1458 (66.5)	
Missing	7 (0.2)	2 (0.2)	5 (0.2)	
Race or ethnicity				0.14
Hispanic	637 (19.8)	193 (18.7)	444 (20.3)	
Non-Hispanic Black	690 (21.4)	249 (24.1)	441 (20.1)	
Non-Hispanic White	596 (18.5)	197 (19.1)	399 (18.2)	
Other	112 (3.5)	34 (3.3)	78 (3.6)	
Missing	1191 (36.9)	361 (35.0)	830 (37.8)	

^*^p-value based on chi-square test comparing ACSC to no ACSC groups

^†^Patient age at most recent encounter reported for patients with multiple encounters

**Table 2:** Unadjusted and Multi-Level Multivariable Logistic Regression of Association Between Sociodemographic Characteristics and Encounter for an ACSC, Adjusted for Age, Sex and Race and Ethnicity

Characteristic	Odds Ratio	*p*-value	Adjusted odds ratio (95% CI)	*p*-value
Age (per 10 years)	1.17 (1.11, 1.23)	< 0.0001	1.16 (1.09, 1.25)	< 0.0001
Female (vs. male)	0.94 (0.81, 1.11)	0.48	0.97 (0.79, 1.18)	0.73
Race and ethnicity^*^				
Hispanic	0.96 (0.75, 1.22)	0.72	0.99 (0.79, 1.27)	0.95
Non-Hispanic Black	1.26 (1.00, 1.58)	0.05	1.27 (1.01, 1.61)	0.042
Other	0.94 (0.62, 1.43)	0.78	0.93 (0.61, 1.42)	0.75

^*^Reference = non-Hispanic White

## DISCUSSION

ACSCs were present in nearly a third of HHC patient encounters. Older and non-Hispanic Black patients had greater odds of having an encounter for an ACSC. Our findings suggest that field-based homeless healthcare teams have the potential to prevent hospitalizations for PEH by identifying and managing these conditions.

This is among the first studies to describe the prevalence of ACSCs among patients treated by a field-based homeless healthcare team. In a prior analysis of hospitalizations among PEH in California, an estimated 9% were due to ACSCs.^2^ As in our analysis, this study revealed disparities by age and race, with older patients and Black patients having higher odds of hospitalization for an ACSC.^2^ This aligns with prior evidence of such disparities in non-homeless populations.^6,7^ Systematic screening and management for ACSCs by field-based homeless healthcare teams may decrease the disproportionate burden of morbidity and hospitalizations experienced by older and Black PEH.

Study limitations include reliance on ICD-10 codes, which may not capture all ACSC diagnoses, large amount of missing race and ethnicity data, which may bias findings, and reliance on a single homeless healthcare team experience, potentially limiting generalizability. Further research is necessary to determine the overall prevalence of ACSCs encountered by field-based homeless healthcare teams across the region and nation and if this care delivery model can contribute to reduced acute care utilization for this vulnerable population.
